# Metastable Evaporation of Molecules from Water Clusters

**DOI:** 10.1021/acs.jpca.4c04728

**Published:** 2024-09-26

**Authors:** Viktoriya Poterya, Andrij Pysanenko, Michal Fárník, Juraj Fedor, Klavs Hansen

**Affiliations:** † J. Heyrovský Institute of Physical Chemistry, v.v.i., Czech Academy of Sciences, Dolejškova 2155/3, 182 23 Prague, Czech Republic; ‡ Center for Joint Quantum Studies and Department of Physics, School of Science, Tianjin University, 92 Weijin Road, Tianjin 300072, China

## Abstract

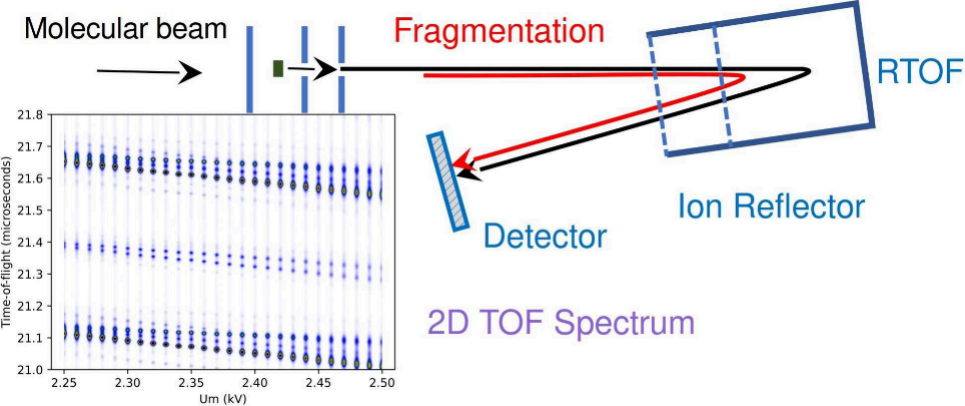

We probe the stability of water clusters by means of their metastable decay probability extracted from two-dimensional reflectron time-of-flight mass spectra. Two different methods are used to ionize and potentially excite the clusters and trigger the evaporation: (i) attachment of electrons with near-zero energies, producing negatively charged  clusters, and (ii) electron impact ionization, producing protonated (H_2_O)_*n*_H^+^ clusters. The electron attachment is a soft ionization and therefore provides information about the size distribution of the neutral clusters in the beam due to a very limited amount of post-ionization loss of water molecules. A dependence of metastable fractions on the conditions of neutral clusters production prior to the electron attachment is reported. For the cations, the higher energy electron impact ionization leads to a more extensive metastable loss of water molecules. The results are discussed in the light of neutral cluster excitation energy distributions and, for negative clusters, also in terms of binding energies. The experiments demonstrate clearly the role of the excess electron vs the excess proton in the two different charge states of the clusters around sizes *N* = 50–55, for which binding energies of the anions are derived from the data.

## Introduction

Water clusters containing hundreds to thousands of water molecules are interesting from both a fundamental point of view and because they provide information about the growth of nanosized droplets that play important roles in, for example, atmospheric and astrophysical contexts. Clusters in a molecular beam constitute isolated finite-size systems that can be probed without the influence of the environment. This is especially important when addressing their thermal properties.^1−6^ Often, the experimental information is obtained mass spectrometrically after the neutral cluster beam is ionized by a vertical trigger, e.g., electron, photon, or ion impact. The ionization typically causes heating of the cluster. In addition to the internal energy with which the clusters are produced in the source, this heating leaves the charged clusters internally excited. The energies frequently exceed the kinetic shift, i.e. the limit beyond which a cluster becomes unstable on the experimental time scale. Part of the excitation energy is then released in delayed evaporation of one or more molecules from the clusters. Here we focus on those decay events that are metastable from the mass spectrometric point of view, i.e., the decays that happen on a time scale on the order of microseconds to tens of microseconds after ionization.

Evaporation events up to the time of detection have a strong influence on the detected mass spectra. This is particularly pronounced for clusters ionized by electron impact with electron energy above the water ionization limit, where protonated water clusters are produced, and a strong fragmentation of neutral clusters occurs immediately after the ionization event.^7,8^ Recent atomistic molecular dynamics simulations^9^ showed a strong fragmentation of charged clusters by prompt events, in the sense that they occur before the end of the simulation time of 1 ns. Earlier experimental studies have shown that evaporation continues at much longer time scales,^10−13^ and, quite remarkably, that the characteristic protonated water cluster mass spectrum even develops on the microsecond time scale, making the process measurable in a mass spectrometer.^10^ Other studies show thermal decays of water clusters with 1/*t* dependent rates, characteristic for decays from broad energy distributions, indicative of a background excitation energy prior to ionization.^13,14^ The systematics associated with this type of decay rate provide the backdrop for the results generated with the high energy ionization in the present experiments.

Attachment of near-zero kinetic energy electrons to water clusters is known to be much less fragmentative.^15^ However, no quantification of the evaporation, manifested for example as a difference between the neutral and anion cluster size distribution, has been reported, and no information about the amount of metastable decay is available.

The experimental method for determining metastable cluster fragmentation by using a reflectron time-of-flight mass spectrometer was pioneered by Schlag and collaborators^16^ and implemented in many studies in the group of Castleman.^17−23^ An extension of this technique, a two-dimensional reflectron time-of-flight mapping of decay products, has been introduced recently for identifying metastable fragments of multiphoton UV photodissociation of simple biomolecular ions.^24^ We used this method for the large clusters studied here and show that it greatly simplifies the assignment of metastable peaks. Their specific traces in 2D maps enable an unambiguous identification even in the presence of numerous overlapping peaks. We use this to detect the metastable evaporation that follows electron attachment and show that it is sensitive to the production conditions of the neutral clusters, primarily to the presence of a cooling gas. On the other hand, the metastable evaporation from the positively charged protonated clusters does not show any significant variation with the production conditions, yielding values that are independent of the cluster source temperature.

## Experiment

### Experimental Setup

The water clusters were generated in a continuous supersonic expansion of water vapor, either pure or seeded with argon or neon buffer gas, which both acted as a cooling agent. The gas was expanded through a conical nozzle (90 μm diameter, 30° full opening angle, 2 mm length), which was attached to the water-containing reservoir located in the source vacuum chamber. The reservoir temperature and nozzle temperature, *T*_R_ and *T*_N_, respectively, were kept constant by separate heating at values *T*_N_ ≥ *T*_R_ to avoid clogging of the nozzle. The conditions used in the different experiments are summarized in Table 1 together with the neutral cluster mean sizes calculated from the expansion conditions according to the modified Hagena formula^25^ (note that the formula has not been derived for coexpansion with a buffer gas).

**Table 1:** Expansion Conditions: *T*_R_ and *T*_N_ Are Reservoir and Nozzle Temperatures, Respectively; *P*_b_ Is the Buffer Gas Backing Pressure; and  Is the Calculated Neutral Cluster Mean Size

	buffer	*P*_b_ (bar)	*T*_R_ (°C)	*T*_N_ (°C)	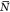
A)	–	–	100	130	50
B)	–	–	130	150	200
C)	Ar	2	100	130	–
D)	Ne	2	100	130	–
E)	–	–	150	160	530

**Figure 1:**
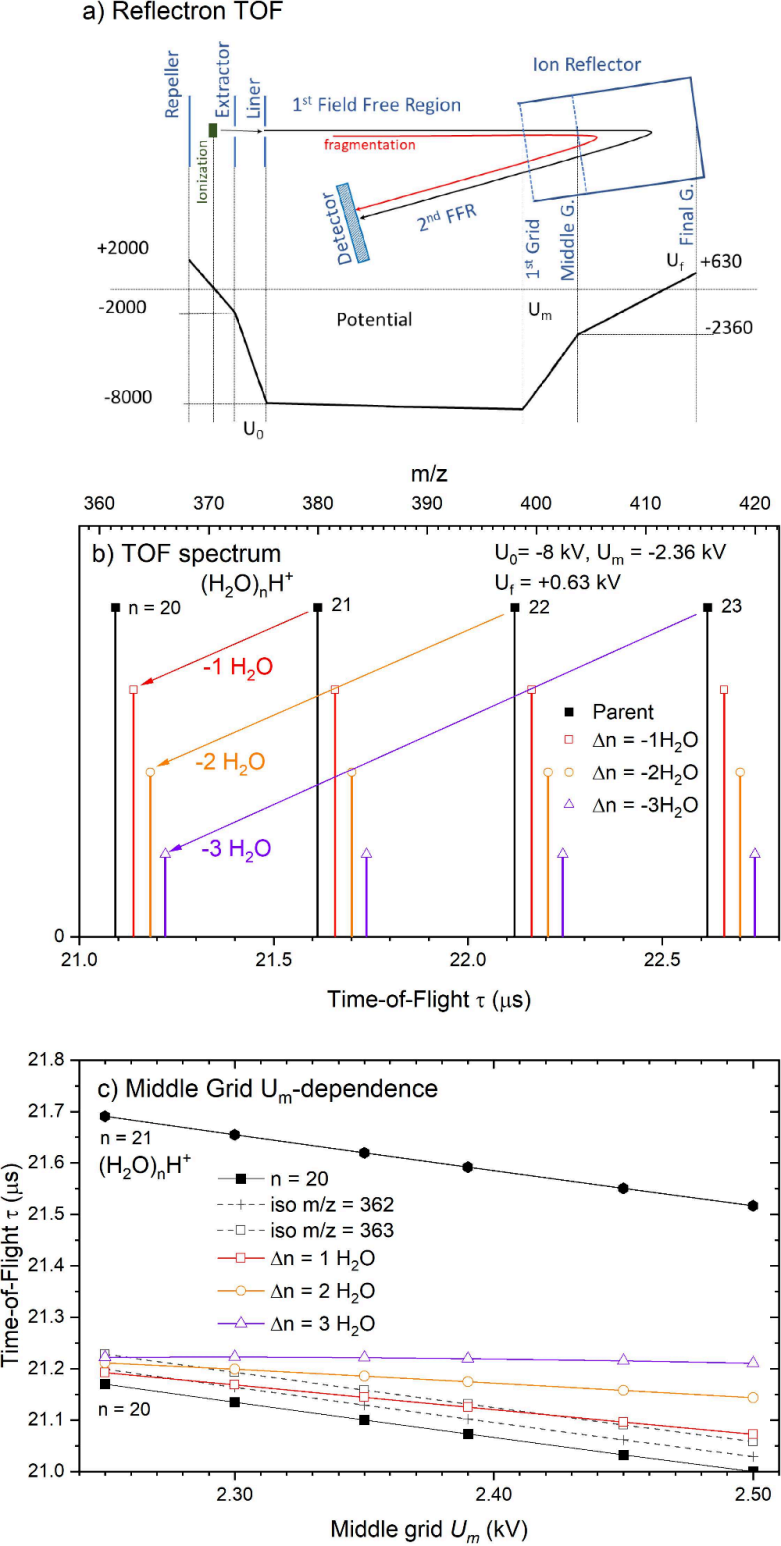
(a) The TOF scheme with the typical potentials in the positive ion mode.(b) Calculated TOF spectrum: the black bars correspond to the peak positions for the parent (H_2_O)_*n*_H^+^, *n* = 20–22, ions; the red, orange and violet bars indicate the positions of the fragments (H_2_O)_*n*′_H^+^ corresponding to the metastable evaporation of 1, 2, and 3 H_2_O molecules, respectively. The height of the bars is arbitrary. (c) Calculated flight times τ as a function of the middle grid voltage *U*_m_ for several parent and fragment ions. The τ(*U*_m_) dependencies for isotopes Δ*m*/*z* = +1, +2 are shown as black dashed lines.

After production, the clusters passed through three differentially pumped vacuum chambers and were then mass analyzed with a reflectron time-of-flight mass spectrometer (TOF) described in refs (26, 27). It can be operated in both positive and negative ion modes, and both were used in the experiments reported here. The clusters were ionized by an electron beam pulsed at 10 kHz. To generate positive ions, the clusters were ionized by 70 eV electrons. The ionization pulse time width was 3 μs. After a delay of 0.1 μs, the ions were extracted from the ionization region by a 4 kV pulsed voltage and further accelerated to 8 keV and injected into the free flight region. In the negative ion mode, the electron energy was set to approximately 1 eV to generate  ions (below 1 eV the electron current of our electron gun becomes too low). The other parameters remained the same as in the positive ion mode, apart from the voltage polarity. After a total flight path of approximately 95 cm in the reflectron TOF, the ions were detected with a microchannel plate detector. Figure 1a shows the schematics of the TOF with typical potentials used in the positive ion mode. The acquisition system is described in ref (27).

The TOF is mounted perpendicularly to the molecular beam axis. Since all the clusters generated in a supersonic expansion attain essentially the same velocity independent of their size, the clusters of different sizes have different momenta in the beam direction. This introduces mass discrimination, i.e., not all cluster sizes will reach the detector. This mass discrimination is mitigated by large extraction and acceleration voltages and by a large area detector (4 cm diameter). Some of the remaining discrimination is compensated by using different deflection voltages. Nevertheless, very broad cluster distributions as the present ones for the large water clusters cannot be recorded completely without some amount of mass discrimination (for a discussion of the mass discrimination in our TOF, see ref (7)). It should thus be kept in mind that the mass spectra shown below are not the neutral cluster size distributions, which are expected to extend to much larger sizes corresponding to the calculated mean neutral cluster sizes in Table 1.

### Determination of Metastable Fragments

The mean size of the generated water clusters (H_2_O)_*N*_ were  50–530, and they were flying for ≈1 ms in ultrahigh vacuum before ionization, as described above. The clusters were ionized centrally between the Repeller and Extractor grids and extracted by the voltages applied to these electrodes (see Figure 1a). Further, they were accelerated to the Liner potential *U*_0_. Then the ions flew through the 1^*st*^ field free region (FFR) into the reflector, where they were first decelerated to the Middle Grid potential (*U*_m_) and then turned around before reaching the Final Grid at potential (*U*_f_). The ions were accelerated again to the free flight potential and flew through the 2^*nd*^ FFR, before reaching the detector.

After ionization, the clusters may have fragmented promptly, i.e., before having moved any appreciable distance in the extraction region. Such prompt fragments appeared in the mass spectrum at the *m*/*z* of the fragmentation product. However, clusters could also evaporate after the completion of the initial acceleration, during their flight time in the 1^*st*^ FFR, i.e., before entry into the reflector. Typically, the ions spent several (tens) of μs in the 1^*st*^ FFR, allowing ample time to decay. Such metastable cluster ions were accelerated in the ion source as one mass (*m*/*z*) but decelerated in the reflector as another fragment mass (*m*′/*z*). Thus, their flight times did not correspond to *m*/*z*, nor to *m*′/*z*.

For example, if the water cluster ion (H_2_O)_*n*_H^+^ was formed in the ion source and extracted into the 1^*st*^ FFR, where a water molecule was evaporated, the resulting decay product (H_2_O)_*n*−1_H^+^ arrived into the detector before the parent (H_2_O)_*n*_H^+^ ion, however, it had a different time-of-flight also from the (H_2_O)_*n*−1_H^+^ ion produced directly in the ion source. The metastable fragment peaks then appeared nominally at intermediate masses. The precise spacing of the metastable fragment and unfragmented peaks could in principle be determined in a computable way by the reflectron fields and the lost mass. However, the unfragmented and metastable fragment ion peaks could be distinguished also experimentally, since their flight times exhibited different dependencies on the applied TOF voltages (namely *U*_m_). Thus, plotting the measured spectra in 2D as a function of the time-of-flight and the middle grid voltage *U*_m_ allows unambiguous determination of the metastable fragments and the stable parent ion peaks, as will be shown below. The use of the reflectron TOF mass spectrometric technique to investigate metastable fragmentation has been described previously on a number of occasions, see for example refs (23, 28) for reviews.

To illustrate the technique we show in Figure 1b) the ion flight times for several (H_2_O)_*n*_H^+^ ions and their fragments. The calculated peak positions for the parent ions (H_2_O)_*n*_H^+^, *n* = 20–22, are indicated by the black bars, and the red, orange, and violet bars indicate the positions of the fragments (H_2_O)_*n*−Δ*n*_H^+^ corresponding to the metastable evaporation of Δ*n* = 1, 2, and 3 molecules, respectively. The height of the bars is chosen arbitrarily.

A series of spectra were recorded with varying values of the middle grid voltage *U*_m_ in order to unambiguously assign the metastable and prompt peaks, and to disentangle peaks in the parts of the spectra where multiple metastable fragmentation peaks and the presence of isotopologue distributions cause congestion. For (H_2_O)_*n*_H^+^, *n* = 20, for example, the latter will give the main peak at *m*/*z* = 361, and minor peaks at *m*/*z* = 362, 363, and 364 with intensities of 1.5%, 4.0% and 0.1%, respectively, of the main peak. The peaks of several percent intensity of the main peak are already comparable with the metastable ion intensities, as will be shown below. Isotope peaks are distinguished from fragment peaks by their different voltage dependencies. The isotope contributions’ τ(*U*_m_) dependencies are parallel to the parent ion dependence, as indicated in Figure 1c) by the dashed lines. The two-dimensional analysis thus provided an unambiguous assignment of the metastable peaks.

## Results and Discussion

### Negatively Charged Clusters—Experimental Results

**Figure 2:**
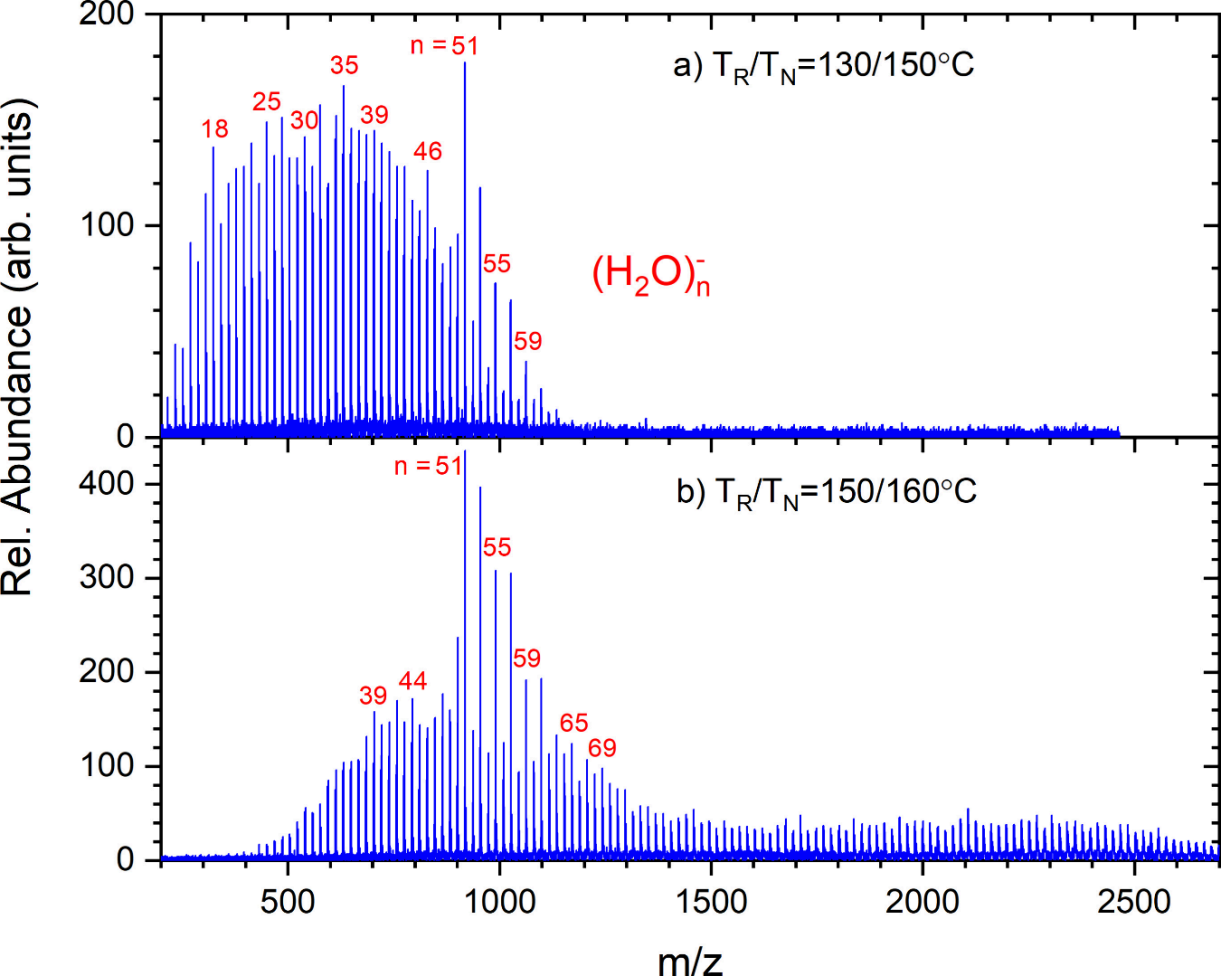
Examples of the mass spectrum of negatively charged water clusters  recorded at 1 eV electron collision energy under the expansion conditions indicated in the frames.

We begin the analysis with negatively charged clusters since these spectra are simpler than the positive ion spectra. It is well-known that the attachment of near-thermal (slow) electrons to water clusters produces  anions, while the attachment of electrons with energies around 7.5 eV causes a deprotonation producing (H_2_O)_*n*_OH^–^ anions.^15^ Here, the energy of the electrons is tuned to produce only the intact  anions. Figure 2 shows two negative ion mass spectra recorded at electron energies of 1 eV but with different source conditions. The top spectrum (a) is recorded with the expansion conditions B in Table 1: pure water vapor expansion at reservoir/nozzle temperature *T*_R_/*T*_N_ = 130/150 °C (corresponding to the calculated mean size  200). A spectrum recorded at the elevated temperatures *T*_R_/*T*_N_ = 150/160 °C ( 530) is shown in panel (b). Further spectra recorded under different conditions in more detailed resolution are shown in Supporting Information.

The most striking features of both spectra are the odd–even oscillations between sizes *N* = 50 and 60. This oscillation has been observed previously,^15^ but it has so far not been analyzed quantitatively. This analysis will be made below, and we first consider the overall shape (envelope) of the spectra. As explained in the experimental section, the perpendicular configuration of the source and the TOF cannot be expected to record the spectrum in the entire mass range without any mass discrimination. The TOF was tuned to a maximum gain for *m*/*z* around 500–1000, and the intensities for smaller and larger masses than this are therefore suppressed. Still, the shift of the mass spectrum toward much larger masses between the two panels of Figure 2 is obvious.

The “softness” of charging the clusters negatively by slow electron attachment can be concluded from purely energetic reasoning. Upon attachment the internal energy of the cluster increases by the electron affinity. This will be similar to the electron vertical detachment energy which has attracted a lot of experimental attention in photoelectron spectroscopy and which is therefore well-known to be between 2 and 2.5 eV for water clusters in the present size range.^29^ With the bulk binding energy of 0.45 eV this corresponds to a loss of 4–5 molecules, provided the kinetic shift does not change significantly, which is not likely to occur by loss of only a few molecules. The internal energy increase caused by electron attachment can thus at most lead to evaporation of a small fraction of the clusters’ masses and therefore does not significantly modify the overall shape of the cluster size distribution. The assumption that the kinetic shift does not change significantly is equivalent to the assumption that the dissociation energy does not decrease upon electron attachment. As the charging energy will tend to increase the dissociation energy, this is a quite reasonable assumption.

The mean cluster sizes calculated from the modified Hagena’s scaling formulas with parameters derived in ref (25) take the values  200 and 530. These parameters were obtained from the mass spectra of photoionized sodium-doped water clusters under the assumption that such ionization method (abbreviated as Na–PI) is nonfragmentative and the mass spectra therefore correspond to the neutral cluster size distribution. Clearly, this is the case also for the current slow-electron attachment, and, similarly as it was suggested for Na–PI,^30^ it can be viewed as a sizer of water clusters.

**Figure 3:**
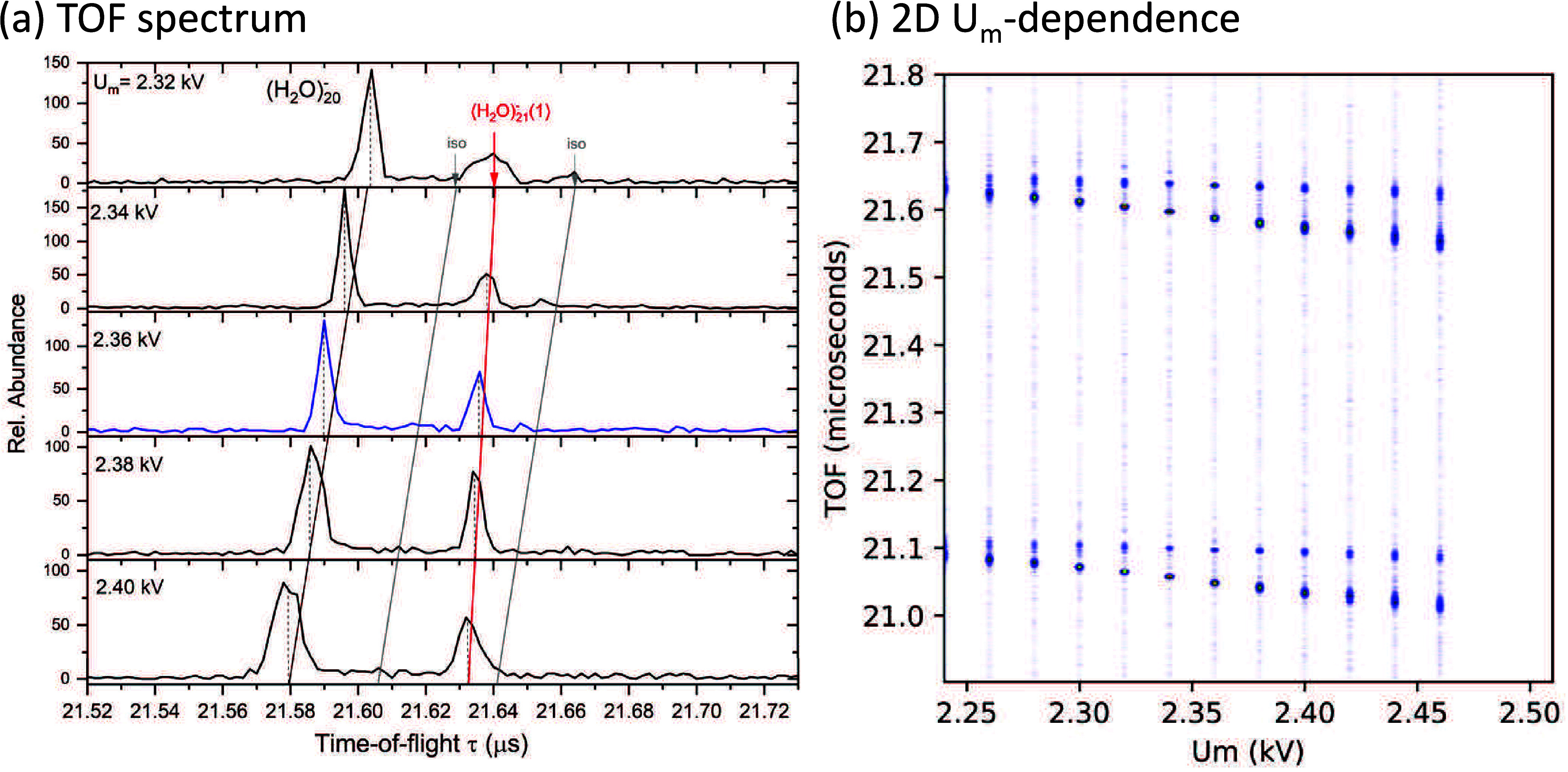
(a) Part of the time-of-flight spectrum around , *n* = 20, as a function of the reflector middle grid voltage *U*_m_. (b) 2D plot of the time-of-flight spectrum around , *n* = 19–20, vs the reflector middle grid voltage *U*_m_.

Figure 3 shows the dependence of the mass spectra on the middle grid voltage, as discussed in the experimental section. In Figure 3a the major TOF peak  shifts with *U*_*m*_ differently than the peak labeled (1) which corresponds to the metastable loss of a water monomer from . We also mark the position of the peaks where the isotopes of the parent ion are expected, even though the S/N ratio is not sufficient to reveal these (they will become relevant in the discussion of positive ions where much higher signals are recorded). The 2D spectrum in Figure 3b reveals immediately the different slopes in the *U*_m_-dependence of the parent and metastable peaks, and broadening of the TOF peaks as *U*_m_ is changed from the mass-focusing condition *U*_m_ = 2.36 kV.

For the intensities of the metastable peaks thus identified we calculated the metastable fraction as
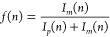
1where *I*_*p*_(*n*) and *I*_*m*_(*n*) are the integrated intensities of the parent peak with *n* water molecules and its corresponding metastable fragments, respectively. For this purpose, the spectra with the optimal resolution settings were used because these provided the best peak-separation-to-width values.

**Figure 4:**
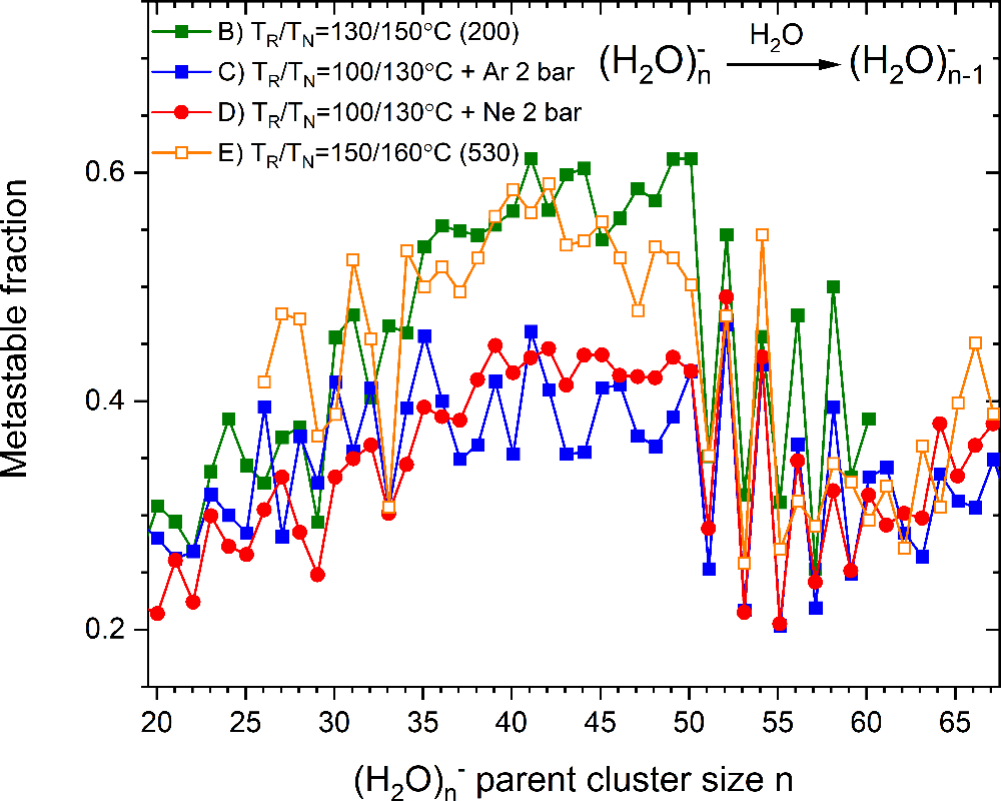
Metastable fraction *f*(*n*) of negatively charged water clusters  produced at different expansion conditions as labeled and summarized in Table 1. The neutral cluster mean size is indicated in parentheses, if available.

Figure 4 shows the metastable fraction *f*(*n*) for different expansion conditions. The expansion conditions are summarized in Table. 1 (the conditions A yield too small clusters to generate any reasonable signal of the negative ions, such data set is thus missing in Figure 4 but will be discussed for the positive ions). The metastable fraction clearly depends on the expansion conditions. The clusters produced in the pure water vapor expansions (B, E) exhibit a significantly higher metastable fraction in the intermediate size region *n* = 35–50 than do those produced in a coexpansion with Ar and Ne and with a lower source temperature (entries C, D in the table).

The expansion conditions clearly have consequences for the vibrational excitation energy distributions of the neutral clusters. Since the electron attachment transfers only a small amount of internal energy into the cluster, different excitation energies prior to the attachment will therefore have a strong influence on the amount of observed metastable fraction. The results in Figure 4 thus suggest that the vibrational energies are higher for the two conditions of neat expansion than for both Ar and Ne coexpansions.

This is only partly in line with the cluster temperature estimates of Becker et al.^31^ of 106 and 93 K for neat expansions B and E, respectively, 41 K for Ar coexpansion C and 100 K for Ne coexpansion D. The present results rather suggest that the vibrational temperature with Ne or Ar present as cooling gas are similar to each other and lower than those of clusters produced in pure water expansions. The present measurements provide a more direct probe of the internal energy of the clusters, and will allow a calibration of the semiempirical method used in ref (31).

When discussing the energies and the temperatures in the expansions, it is important to understand that the neat water vapor expansions may simply show the upper limit of possible metastable fragmentation on the time scale it is measured. Evaporation events prior to mass selection in the acceleration in the TOF are possible and are obviously outside the dynamic range of the mass spectrometer. It is likely that the two high value curves in Figure 4 reach this upper limit, given their close similarity and the similarity with the size dependence of previously measured metastable fractions.^13^ The conclusion is therefore that the two neat expansion conditions produce the same energy content in the clusters at the time of mass selection.

We now address the odd–even oscillations in the mass spectrum in the range *n* = 50–60. They are seen in the mass spectra in Figure 2 as exceptionally intense odd peaks *n* = 51, 53, 55, 57, 59, and the even peaks suppressed, not only compared with the odd mass peaks but also compared with even peaks just below and above the 50–60 range. The metastable fractions (Figure 4) of odd peaks are correspondingly low, and those of even peaks are high. The odd magic numbers in the anion mass spectra were observed previously both in experiments with electron attachment^15^ and with electron transfer from high-Rydberg rare gas atoms.^32^ In order to explain the presence of the even magic numbers seen in ref (29), the assumption that the electron is taking the place of a single water molecule must be added to a geometric interpretation of the odd magic numbers seen here. The difference between the experiments is ascribed by the authors of ref (29) to a different past-production processing of the clusters. The geometric nature of the stability is supported by the fact that also clusters containing a single foreign molecule show odd magic numbers,^33^ as do deprotonated clusters, if the OH^–^ is included in the counting of the water molecules.^14^

Adding to the question of stabilities is the fact that the water clusters absorb ambient radiation. Lifetime measurements of *n* = 52–56 cationic clusters showed clear odd–even effects with odd clusters having longest lifetimes, suggesting higher stabilities.^34^ Obviously the absorbance of the clusters may also have a size dependence and these results are unfortunately not directly applicable to interpret the present experiments.

### Negatively Charged Clusters—Abundances in Terms of Evaporative Ensemble Dynamics

Given the similarity of the odd–even abundances reported here and those observed previously in the literature, we will cautiously assume that an evaporative equilibrium was established in our experiments, at least for the clusters below *n* ≈ 25 and above *n* = 50. This is corroborated by a comparison of the metastable fraction of the neat and seeded expansion spectra. The ratio of the two is a smoothly varying function of size, with a value of unity around 25 and above 50, and with a broad dip centered around *n* = 40–50. In particular, there is little sign of a deviation from unity in the ratio of the otherwise strong odd–even oscillations between 50 and 60. We will use the neat expansion spectra as the input of an evaporative ensemble calculation of the odd–even oscillation of the dissociation energies.

The relevant equations for this purpose have been derived previously in ref (35). A more detailed exposition can be found in ref (36) and we will refer readers to these references for details. The first step in the analysis is to determine if the clusters here belong in the small or the large cluster category. In this context, the question is decided by the magnitude of the heat capacity. With a size of around *n* = 55, the heat capacity is around 300 *k*_B_, found by reading off the number from the curve *C*_*v*_ vs *n* in ref (13). This should be compared with the value of , where ω = 8.7 × 10^17^ s^–1^ is the frequency factor for the statistical rate constant,^14^ and *t* is the mass selection time, i.e., the time between ionization and the time the unfragmented clusters can be considered accelerated. It is basically the time it takes to go through half the acceleration energy. The frequency factor is calculated from the bulk vapor pressure and adjusted to a geometric capture cross section for the inverse process. This gives *G*^2^ = 630, i.e. a factor of 2 larger than the heat capacity, and this places the clusters studied here in the small cluster category. We do not expect that the precise charge state nor the temperature variations of the heat capacities will change this conclusion.^14^

We can therefore use the equation derived in ref (35) for the abundances *I*:
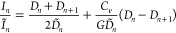
2The heat capacity *C*_*v*_ is measured in units of *k*_B_ here. The  is the liquid drop dissociation energy, i.e., the binding energy of a sphere with bulk cohesive energy and surface tension (this value for our clusters is 0.45 eV).  is the smooth part of *I*_*n*_ as a function of size. It is found as a smoothly varying function with a procedure given in, e.g., ref (37).

**Figure 5:**
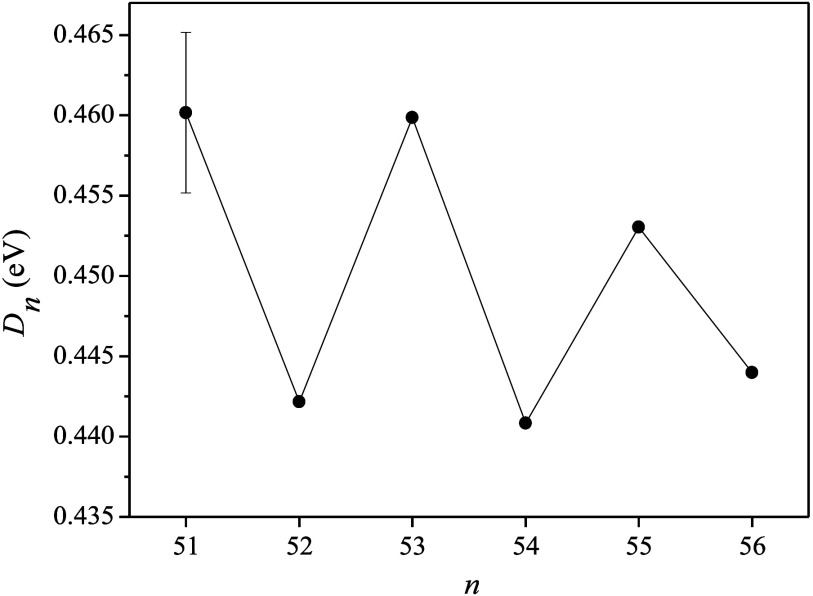
Dissociation energy of clusters *n* = 51–56. The intensities refer to the time of entry into the reflectron, i.e., all fragment peaks are counted as belonging to the product. The values are calculated from eq 3 and setting ,  for *n* odd. The error bar is representative for all points and estimated from the spread in values in Figure 4 and includes systematic errors.

In the present case we can approximate the relation as
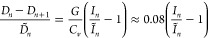
3where the ratio *G*/*C*_v_ has approximately been set to the constant 0.08 based on the values quoted above. Figure 5 shows the values of the dissociation energies of *n* = 51–56 derived this way for spectrum E) in Figure 4, after multiplication by a liquid drop energy of 0.45 eV. The main point in the figure is the magnitude of the oscillations. The error bar gives a conservative estimate of the robustness of the odd–even effects in the dissociation energies. The very small effect is a direct consequence of the magnitude of the factor *C*_*v*_/*G* ≈ 12, which provides a strong amplification of the odd–even effect in the abundance spectra. The absolute value is contingent upon the reliability of the liquid drop model. Any change in the binding energy will simply shift the curve up or down on the energy axis with a constant factor in proportion.

In a theoretical study,^38^ Kazachenko and Thakkar used different potential energy models to derive the binding energies in clusters of *n* ≤ 55 water molecules. It is interesting to note that they observed the oscillations in monomer binding energies for *n* = 51 to 55 quite similar to the ones in Figure 5. Although, the theoretical binding energies for all applied force fields are below our present values derived from the experiment, the qualitative agreement is quite encouraging.

### Positively Charged Clusters

**Figure 6:**
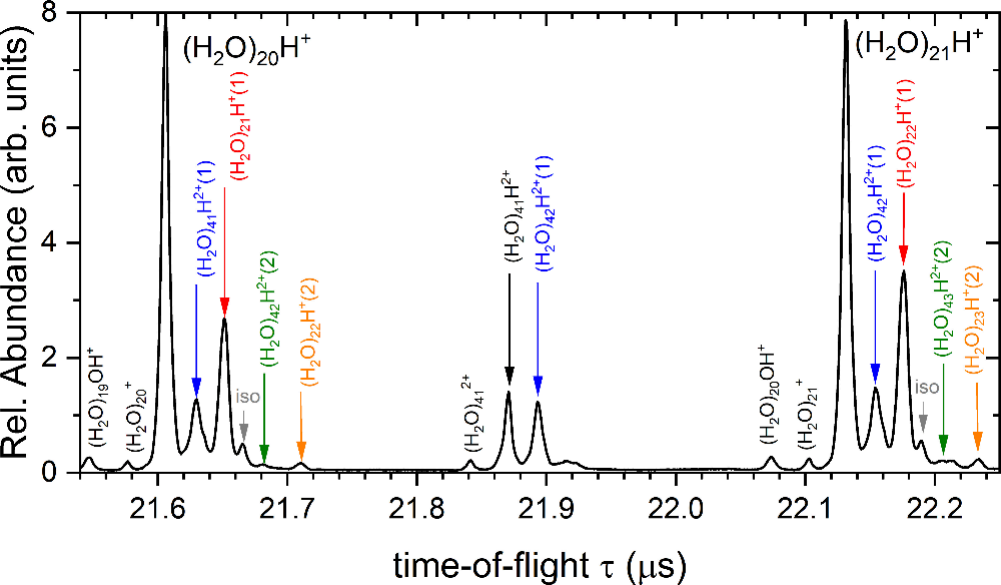
Section of the mass spectrum illustrating the contribution of the doubly charged (H_2_O)_*n*_H^2+^ cluster ions to the metastable fragment ion signal. The labels are as in Figure 7.

A section of the positive mass spectrum recorded at 70 eV electron energy is shown in Figure 6. The final assignments of the peaks marked by colored arrows follow from the analysis presented below. The spectrum is dominated by protonated ions (H_2_O)_*n*_H^+^, in agreement with all previous studies. More than 30 times weaker peaks are seen for  and (H_2_O)_*n*−1_OH^+^. Additionally, doubly charged ions, and isotopic and metastable peaks are present.

**Figure 7:**
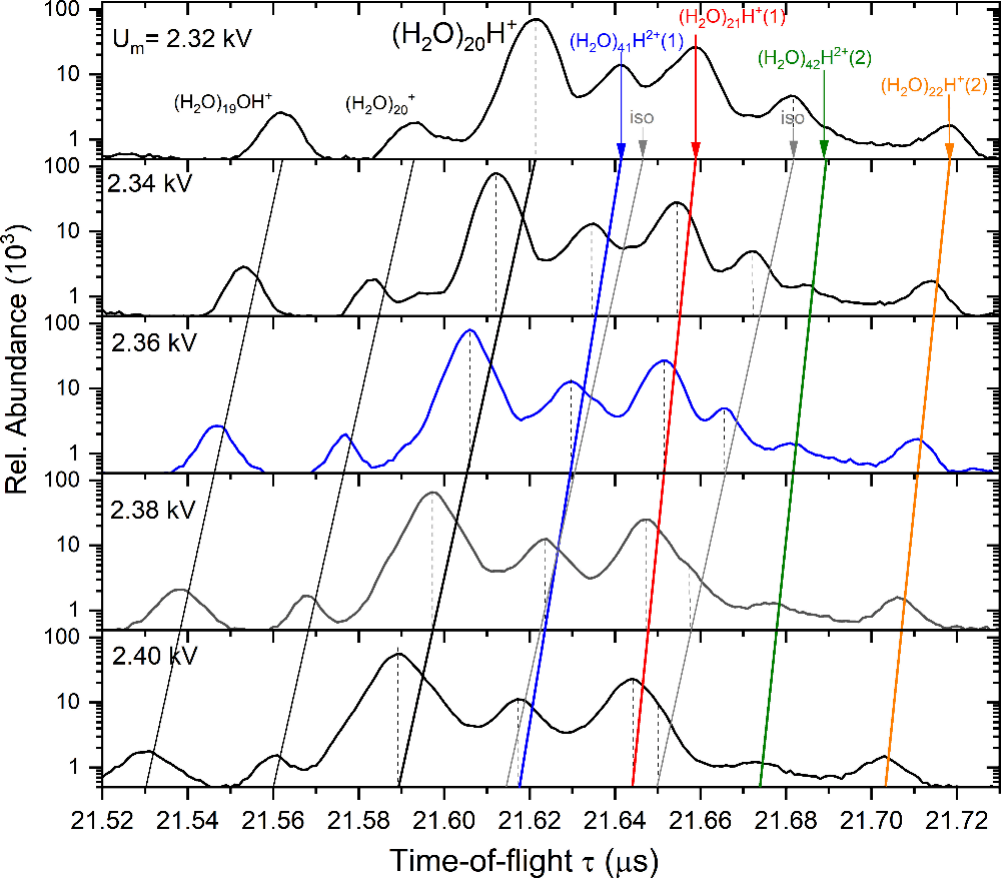
Detailed analysis of the part of the time-of-flight spectrum around (H_2_O)_*n*_H^+^, *n* = 20, ion as a function of the reflectron middle grid voltage *U*_m_.

The dependence on the *U*_m_ voltage, shown in Figure 7 on a semilogarithmic scale, is thus considerably more congested than for the negative ions. The peaks overlap even at the focusing condition (*U*_m_ = 2.36 kV) and they broaden for other values. The *U*_m_ dependences of the weak peaks of  and (H_2_O)_19_OH^+^ are parallel to the major peak of (H_2_O)_20_H^+^, which is connected by a black line across the frames. We also indicate the expected positions (short gray arrows) of the two isotopologue peaks with the expected total abundances of 1.5% and 4.0%. The former is weak and hidden in the congested region of the spectra, while latter is clearly visible at *U*_m_ ≤ 2.36 kV and merges with the preceding peak at higher voltages.

There are several mass peaks, indicated by colored arrows, for which the *U*_m_-dependences are not parallel to the (H_2_O)_20_H^+^ black line. These are assigned to the metastable ions. First, the major metastable peak labeled by red arrow as (H_2_O)_21_H^+^(1) corresponds to the evaporation of one H_2_O molecule from (H_2_O)_21_H^+^. The orange labeled (H_2_O)_22_H^+^(2) peak corresponds to the (H_2_O)_22_H^+^ → (H_2_O)_20_H^+^ + 2·H_2_O evaporation.

Doubly charged ions (H_2_O)_*n*_H^2+^ appear halfway between the singly charged ion peaks for *n* odd (see Figure 6) and coincide with the singly charged ions with identical *m*/*z* for *n* even. Their metastable fragments will interfere with those produced in the metastable fragmentation of the singly charged ions. For example, the (H_2_O)_41_H^2+^ ion appears after one H_2_O molecule evaporation as (H_2_O)_41_H^2+^(1) (blue arrow) half way between the (H_2_O)_20_H^+^ parent ion and the first metastable (H_2_O)_21_H^+^(1) peak. The peak corresponding to two H_2_O molecules evaporation from the (H_2_O)_42_H^2+^ ion is also recognizable in the spectra indicated as (H_2_O)_42_H^2+^(2) (green arrow).

**Figure 8:**
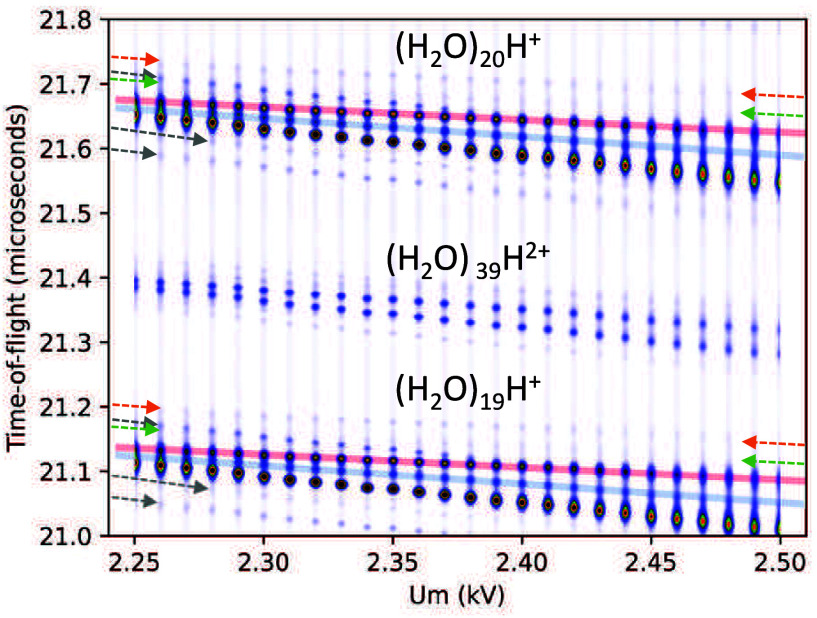
A 2D plot of the time-of-flight spectrum around (H_2_O)_*n*_H^+^, *n* = 19–20, ion vs the reflectron middle grid voltage *U*_m_. The main peaks are indicated by lines with color coding corresponding to Figure 7. Orange and green lines used in Figure 7 denote weak peaks, thus only arrows pointing to both end of the straight lines are used here not to obscure the weak dots. The gray arrow above the green corresponds to the second isotopologue that merges with the green and red lines at higher *U*_m_. Gray arrows below the row of the main peaks correspond to the  and (H_2_O)_*n*−1_OH^+^ fragments.

Figure 8 shows the 2D plot of the *U*_m_ dependence of the time-of-flight spectrum. The peaks are highlighted by lines using the same color coding as in Figure 7. The 2D plot also shows the doubly charged ions and the bottom row corresponding to the *n* = 19 ion peaks.

**Figure 9:**
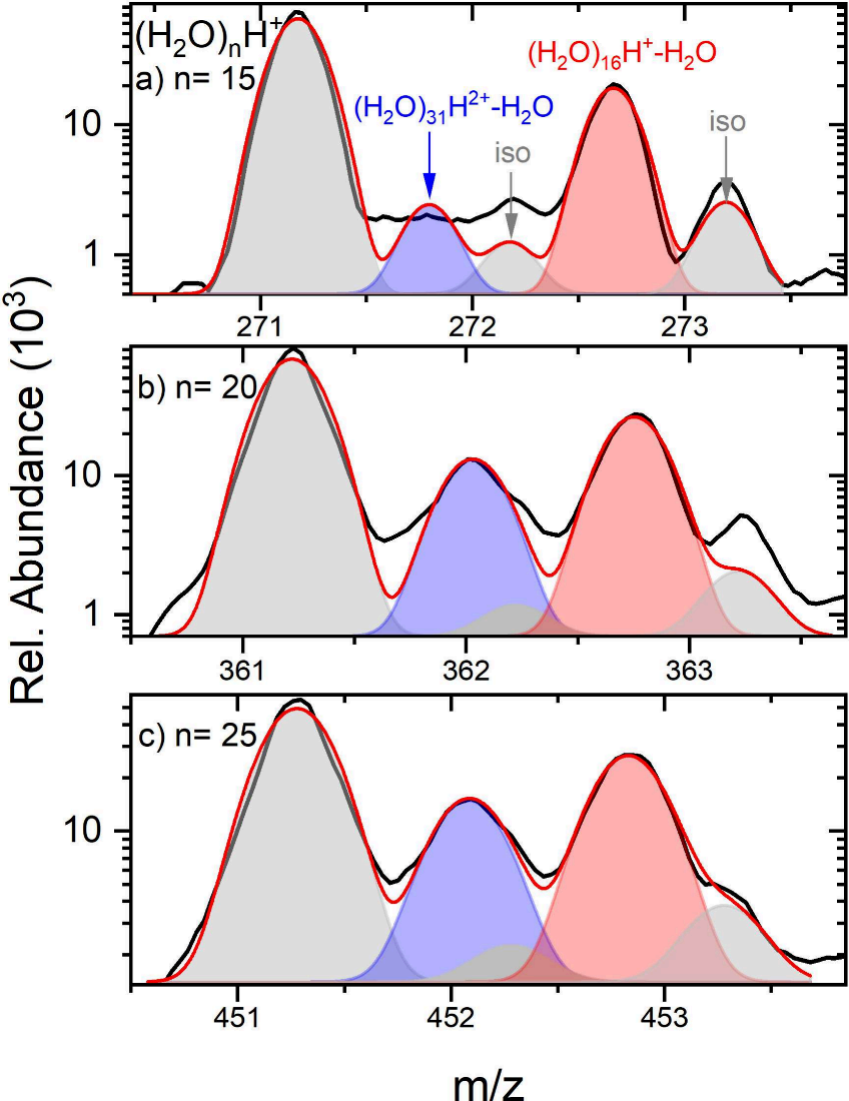
Gaussian fits of the individual contributions to the (H_2_O)_*n*_H^+^ mass peaks for a) *n* = 15, b) 20, and c) 25. The gray peaks correspond to the parent ions and its isotope contributions. The red peaks correspond to the metastable fragments after one H_2_O evaporation from (H_2_O)_*n*+1_H^+^, and the blue peaks correspond to the metastable doubly charged fragments (H_2_O)_2*n*_H^2+^.

Given the complicated nature of the spectra, we fitted them with contributions of individual peaks assuming Gaussian shapes, as illustrated in Figure 9 for (H_2_O)_*n*_H^+^ mass peaks *n* = 15 a), 20 b), and 25 c). The major parent ion peak (gray) is first fitted alone and the corresponding isotope peaks (also gray) are then fixed, since their position and abundance are derived from the major peak. The red and blue peaks correspond to the metastable fragments after evaporation of a single H_2_O molecule from the corresponding singly and doubly charged clusters, respectively. From the fits of these peaks the metastable fractions were determined.

There are a few points about evaluating the metastable fraction which should be mentioned. First, the isotope contributions of the metastable peaks are neglected due to their low intensity. Second, and more importantly, the doubly charged (H_2_O)_2*n*_H^2+^ ions contribute to the (H_2_O)_*n*_H^+^ peaks, as mentioned above. In the parent ions, this contribution cannot be disentangled, since the peaks overlap. Nevertheless, we can estimate this contribution by interpolating between the odd-*n* (H_2_O)_*n*_H^2+^ peaks, which are located between the singly charged ion peaks. Finally, although the peaks corresponding to the evaporation of two water molecules were identified in the spectra, their low intensities and the spectral congestion did not allow to evaluate reliably their metastable fractions.

**Figure 10:**
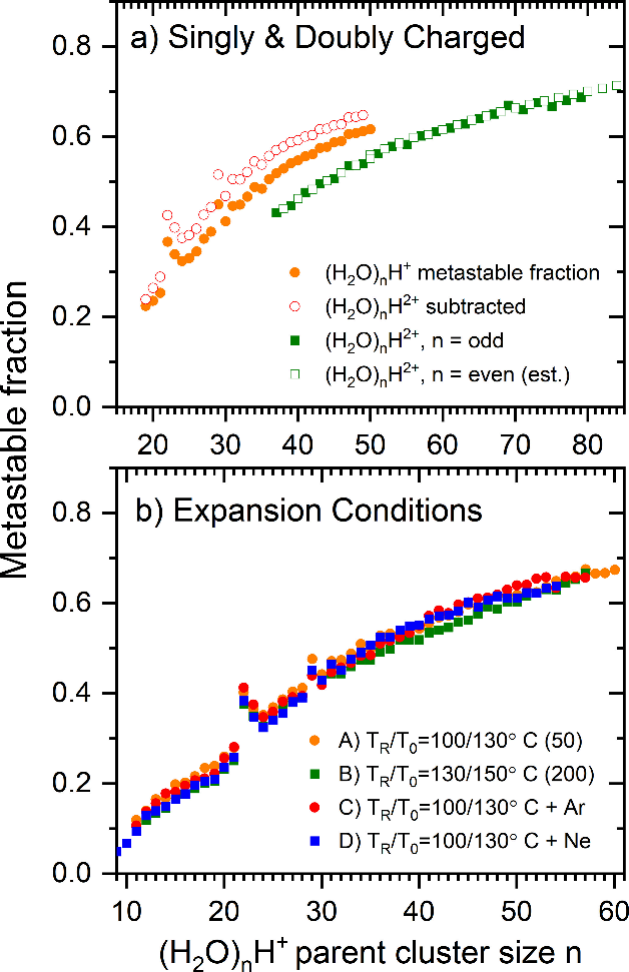
a) The metastable fraction of (H_2_O)_*n*_H^+^ as a function of the number of water molecules in the parent ion (filled orange circles). The open red circles show the values corrected by subtraction of the doubly charged cluster intensity from the parent. The solid green squares show the metastable fraction of the doubly charged (H_2_O)_*n*_H^2+^ cluster ions for odd-*n*; the even-*n* values (open squares) were estimated using interpolated values for parent ions. See the text for the details of the procedure. b) The metastable fraction of (H_2_O)_*n*_H^+^ ions for different expansion conditions as indicated in the legend.

The metastable fractions are plotted in Figure 10 as a function of the number of water molecules in the parent ions. Their general shape corresponds to earlier reports obtained by different (hard) ionization methods.^11,22,39,40^ The fractions for the singly charged ions are systematically higher than those for the doubly charged ones. In Figure 10b), we have also evaluated the metastable fractions at the various expansion conditions listed in Table 1 (for simplicity, these were not corrected for the doubly charged cluster contribution). In strong contrast to the negative ion metastable fractions, the positive ions show essentially no dependence on the production conditions. Clearly, the much higher excitation energy and the concomitant stronger fragmentation of the positive ionization process^7^ more efficiently deletes the information on the initial temperature of these ions’ neutral precursors than for the anions. At all experimental conditions, there is a local maximum for *n* = 22 (*n* is the parent ion size). This is associated with the low abundance of this cluster just above the *n* = 21 magic number.

These observations are known from previous measurements of protonated water clusters. We point out that the variations in metastable fractions is mainly due to the normalization by the total (initial) parent intensity. The variations are essentially inversely proportional to these intensities. Hence a low metastable fraction of cluster *n* is simply due to the *stability* of that cluster and *vice versa*.

### Systematics of Metastable Decay of Protonated Clusters

The metastable fraction shown in Figure 10 clearly exhibit some systematics. The general increase with size is commonly observed in evaporative cluster studies. It has been traced to the heat capacity of the clusters (cf. eq 3). The connection between the metastable fraction and the heat capacity has been used previously to determine values for mass selected protonated and deprotonated water clusters in ref (13), to which we refer the reader for a derivation. The values were found to be close to linear with size, with a slope of 6–8 *k*_B_ per molecule. As the cluster sources are different in that study and the present, it is of interest to examine the results in this light.

The theory for this purpose is given in ref (36) and will not be rederived here. The analysis will apply some simplifications. Binding energies are all set equal, and heat capacities are set proportional to size but with an offset that has a known origin which basically accounts for the translational and rotational degrees of freedom. The linearity means that some size dependent variations will be beyond the power of the analysis, for example the large value for *n* = 22.

**Figure 11:**
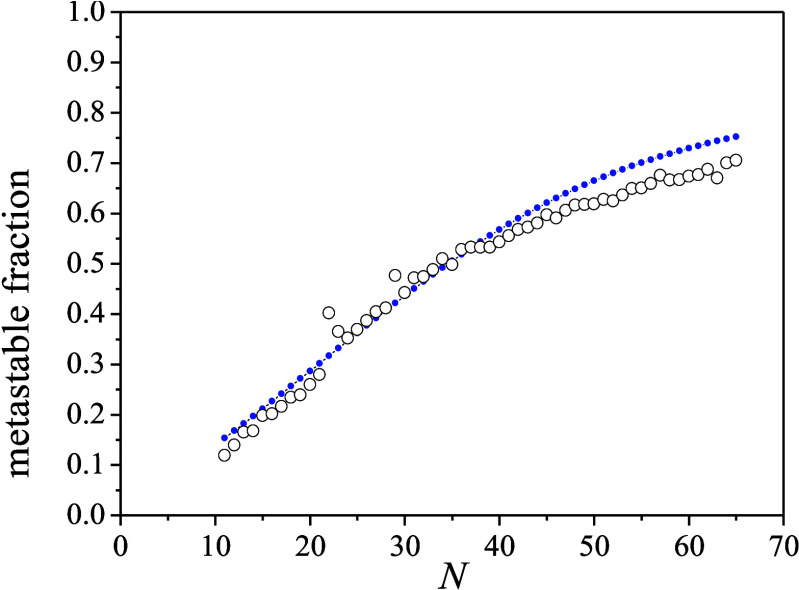
Calculated metastable fraction with the equations and numbers given in the main text. The experimental data (open circles) are the set C) from Figure 10b).

The resulting equation for the metastable fraction of cluster size *n*, *f*_*n*_ is given in a compact form as^36^
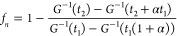
4where *G* is a function of time *G*(*t*) ≡ ln(*ωt*), and ω is used with a size dependence here, calculated from bulk vapor pressure data to
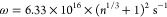
5The two times *t*_1_ and *t*_2_ in eq 4 are the time elapsed after ionization and mass selection time and the time from ionization to entry into the reflectron, respectively. The first time is close to the time it takes to traverse half the initial acceleration field in the TOF as outlined already above. Both times vary with the square root of the mass. The remaining parameter α is defined as the ratio of the two rate constants
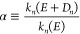
6where the energy is defined by the relation *k*_*n*_(*E*) = 1/*t*_1_. With a simple Arrhenius-type expression, *k*_*n*_(*E*) = ω exp(−*D*_*n*_*C*_*n*_/(*E* + *E*_0_)), and ignoring the difference between *n* and *n* + 1 it is possible to express the parameter as
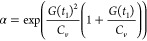
7As before, the heat capacity is expressed in units of *k*_B_. We use the values *C*_v_ = *c*·(*n* – 4), where *c* = 6 to be specific. The parameter *c* is thus the heat capacity per molecule, i.e., *c* ≡ d*C*_v,*n*_/d*n*, in units of *k*_B_. The term −4 accounts for rotational and translational degrees of freedom, as well as the fact that product and reactant are different sizes. With this, the entire curve of metastable fractions vs size is determined. A comparison with one of the measurements shown in Figure 10 is given in Figure 11. The agreement between experiment and theory is seen to be very good, confirming the assumption of an ensemble of clusters that have undergone one or more evaporations before mass selection. An analysis of the abundances of the cations in a reflectron TOF was performed in ref (41). As abundances and metastable fragments are closely related in an evaporative ensemble, the analysis of the metastable decay of the cations in terms of binding energies will not be done here.

## Conclusions

In summary, we have experimentally examined the metastable evaporation of molecules from water using two-dimensional TOF mapping of the decay products. This technique enabled the unambiguous identification of metastable peaks even in the presence of numerous overlapping peaks caused by isotopologues and multiply charged fragments.

The abundances of negative  clusters, produced by a slow-electron attachment, reflect the overall size distribution in the neutral beam since the electron attachment leads to only a small internal energy increase. This also causes the metastable fraction of negative clusters to be sensitive to the expansion conditions at which the neutral clusters were produced. Strong odd–even oscillations are observed both in the mass spectra and in the metastable fractions, for sizes between 50 and 60 molecules. Using a statistical model of the evaporative ensemble dynamics we determine the cluster binding energies for the first time and show that the large variations in abundances are caused by very small variations in dissociation energies.

When the clusters are positively charged by 70 eV electron impact producing protonated clusters, the ionization is much more fragmentative than for the anions and the resulting metastable fractions are insensitive to the production conditions of the neutral clusters. A description of the metastable fractions in terms of broad energy distributions reproduces the experimental behavior very well.

Here, we summarize the novelty of the present work: (i) there had been no quantification of metastable decay in these systems prior to this work; (ii) whereas metastable decay of protonated water clusters had been investigated previously, the present work offers more detailed and more precise data, derived from the employed experimental technique of 2D-mass spectrometry; (iii) the synergy of the present experiment and theory allowed the determination of the cluster binding energies for the negative ion spectra; and (iv) especially for the protonated clusters the symbiosis between the accurate data and the theory provides the key to understanding the cluster fragmentation behavior upon energy deposition.

The protonated water cluster metastable decay after electron ionization exhibits essentially no dependence on the cluster source conditions, i.e., on the original internal cluster temperature, due to relatively large amount of energy deposited into the cluster upon the ionization. In contrast, in the present study the metastable decay of the negatively charged clusters is sensitive to the cluster preparation method, i.e., the electron attachment at low electron energies is a promising method to determine the neutral cluster size and potentially even temperature determination.

## Supporting Information Available

Detailed mass spectra of negatively charged  cluster ions measured under different expansion conditions (PDF)

## References

[ref1] Hock C.; Schmidt M.; Kuhnen R.; Bartels C.; Ma L.; Haberland H.; v.Issendorff B. Calorimetric Observation of the Melting of Free Water Nanoparticles at Cryogenic Temperatures. Phys. Rev. Lett. 2009, 103, 073401 10.1103/PhysRevLett.103.073401. 19792643

[ref2] Schmidt M.; von Issendorff B. Gas-Phase Calorimetry of Protonated Water Clusters. J. Chem. Phys. 2012, 136, 164307 10.1063/1.4705266. 22559482

[ref3] Pradzynski C. C.; Forck R. M.; Zeuch T.; Slavíček P.; Buck U. A Fully Size-Resolved Perspective on the Crystallization of Water Clusters. Science 2012, 337, 1529–1532. 10.1126/science.1225468. 22997336

[ref4] Buck U.; Pradzynski C. C.; Zeuch T.; Dieterich J. M.; Hartke B. A Size Resolved Investigation of Large Water Clusters. Phys. Chem. Chem. Phys. 2014, 16, 6859–6871. 10.1039/c3cp55185g. 24603719

[ref5] Gimelshein N.; Gimelshein S.; Pradzynski C. C.; Zeuch T.; Buck U. The Temperature and Size Distribution of Large Water Clusters from a Non-Equilibrium Model. J. Chem. Phys. 2015, 142, 244305 10.1063/1.4922312. 26133426

[ref6] Moberg D. R.; Becker D.; Dierking C. W.; Zurheide F.; Bandow B.; Buck U.; Hudait A.; Molinero V.; Paesani F.; Zeuch T. The End of Ice I. Proc. Natl. Acad. Sci. U.S.A. 2019, 116, 24413–24419. 10.1073/pnas.1914254116. 31685641 PMC6900515

[ref7] Lengyel J.; Pysanenko A.; Poterya V.; Kočišek J.; Fárník M. Extensive Water Cluster Fragmentation After Low Energy Electron Ionization. Chem. Phys. Lett. 2014, 612, 256–261. 10.1016/j.cplett.2014.08.038.

[ref8] Huang C.; Kresin V. V.; Pysanenko A.; Fárník M. Water Cluster Fragmentation Probed by Pickup Experiments. J. Chem. Phys. 2016, 145, 104304 10.1063/1.4962220. 27634257

[ref9] Suchan J.; Kolafa J.; Slavíček P. Electron-Induced Fragmentation of Water Droplets: Simulation Study. J. Chem. Phys. 2022, 156, 144303 10.1063/5.0088591. 35428398

[ref10] Echt O.; Kreisle D.; Knapp M.; Recknagel E. Evolution of “Magic Numbers” in Mass Spectra of Clusters after Ionization. Chem. Phys. Lett. 1984, 108, 401 10.1016/0009-2614(84)85215-X.

[ref11] Belau L.; Wilson K. R.; Leone S. R.; Ahmed M. Vacuum Ultraviolet (VUV) Photoionization of Small Water Clusters. J. Phys. Chem. A 2007, 111, 10075–10083. 10.1021/jp075263v. 17715907

[ref12] Andersson P. U.; Ryding M. J.; Sekiguchi O.; Uggerud E. Isotope Exchange and Structural Rearrangements in Reactions between Size-Selected Ionic Water Clusters, H_3_O^+^(H_2_O)(n) and NH${}_{4}^+$(H_2_O)(n), and D_2_O. Phys. Chem. Chem. Phys. 2008, 10, 6127–6134. 10.1039/b804584d. 18846302

[ref13] Sundén A.; Støchkel K.; Panja S.; Kadhane U.; Hvelplund P.; Nielsen S. B.; Zettergren H.; Dynefors B.; Hansen K. Heat Capacities of Freely Evaporating Charged Water Clusters. J. Chem. Phys. 2009, 130, 224308 10.1063/1.3149784. 19530769

[ref14] Hansen K.; Andersson P.; Uggerud E. Activation Energies for Evaporation from Protonated and Deprotonated Water Clusters from Mass Spectra. J. Chem. Phys. 2009, 131, 124303 10.1063/1.3230111. 19791877

[ref15] Knapp M.; Echt O.; Kreisle D.; Recknagel E. Electron Attachment to Water Clusters under Collision-Free Conditions. J. Phys. Chem. 1987, 91, 2601–2607. 10.1021/j100294a031.

[ref16] Kühlewind H.; Neusser H.; Schlag E. Metastable Fragment Ions in Multi-Photon Time-of-Flight Mass Spectrometry: Decay Channels of the Benzene Cation. Int. J. Mass Spectrom. Ion Phys. 1983, 51, 255–265. 10.1016/0020-7381(83)85011-6.

[ref17] Echt O.; Dao P. D.; Morgan S.; Castleman A. W. Multiphoton Ionization of Ammonia Clusters and the Dissociation Dynamics of Protonated Cluster Ions. J. Chem. Phys. 1985, 82, 4076 10.1063/1.448849.

[ref18] Morgan S.; Castleman J. A. W. Evidence of Delayed Internal Ion–Molecule Reactions Following the Multiphoton Ionization of Clusters: Variation in Reaction Channels in Methanol with Degree of Solvation. J. Am. Chem. Soc. 1987, 109, 2867–287. 10.1021/ja00244a001.

[ref19] Morgan S.; Castleman A. W. Dissociation Dynamics of Methanol Clusters Following Multiphoton Ionization. J. Phys. Chem. 1989, 93, 4544 10.1021/j100348a031.

[ref20] Morgan S.; Keesee R. G.; Castleman A. W. Reactions of Methanol Clusters following Multiphoton Ionization. J. Am. Chem. Soc. 1989, 111, 3841–3845. 10.1021/ja00193a014.

[ref21] Wei S. Q.; Tzeng W. B.; Castleman A. W. Dissociation Dynamics: Measurements of Decay Fractions of Metastable Ammonia Cluster Ions. J. Chem. Phys. 1990, 93, 2506–2512. 10.1063/1.459033.

[ref22] Shi Z.; Ford J. V.; Wei S.; Castleman A. W. Water Clusters: Contributions of Binding Energy and Entropy to Stability. J. Chem. Phys. 1993, 99, 8009–8015. 10.1063/1.465678.

[ref23] Wei S. Q.; Castleman A. W. Using Reflectron TOFMS Techniques to Investigate Cluster Dynamics and Bonding. Int. J. Mass Spectrom. Ion Processes 1994, 131, 233–264. 10.1016/0168-1176(93)03886-Q.

[ref24] Bockova J.; Rebelo A.; Ryszka M.; Pandey R.; da Fonseca Cunha T.; Limao-Vieira P.; Mason N.; Poully J.; Eden S. Mapping the Complex Metastable Fragmentation Pathways of Excited 3-Aminophenol. Int. J. Mass. Spectrom 2019, 442, 95–101. 10.1016/j.ijms.2019.05.006.

[ref25] Bobbert C.; Schütte S.; Steinbach C.; Buck U. Fragmentation and Reliable Size Distributions of Large Ammonia and Water Clusters. Eur. Phys. J. D 2002, 19, 183–192. 10.1140/epjd/e20020070.

[ref26] Lengyel J.; Pysanenko A.; Kočišek J.; Poterya V.; Pradzynski C.; Zeuch T.; Slavíček P.; Fárník M. Nucleation of Mixed Nitric Acid-Water Ice Nanoparticles in Molecular Beams that Starts with a HNO_3_ Molecule. J. Phys. Chem. Lett. 2012, 3, 3096–3109. 10.1021/jz3013886. 26296012

[ref27] Kočišek J.; Lengyel J.; Fárník M. Ionization of Large Homogeneous and Heterogeneous Clusters Generated in Acetylene-Ar Expansions: Cluster Ion Polymerization. J. Chem. Phys. 2013, 138, 124306 10.1063/1.4796262. 23556722

[ref28] Boesl U. Time-of-Flight Mass Spectrometry: Introduction to the Basics. Mass Spectrom. Rev. 2017, 36, 86–109. 10.1002/mas.21520. 27859457

[ref29] Ma L.; Majer K.; Chirot F.; von Issendorff B. Low Temperature Photoelectron Spectra of Water Cluster Anions. J. Chem. Phys. 2009, 131, 144303 10.1063/1.3245859. 19831437

[ref30] Yoder B. L.; Litman J. H.; Forysinski P. W.; Corbett J. L.; Signorell R. Sizer for Neutral Weakly Bound Ultrafine Aerosol Particles Based on Sodim Doping and Mass Spectrometric Detection. J. Phys. Chem. Lett. 2011, 2, 2623–2628. 10.1021/jz201086v.

[ref31] Becker D.; Dierking C. W.; Suchan J.; Zurheide F.; Lengyel J.; Fárník M.; Slavíček P.; Buck U.; Zeuch T. Temperature Evolution in IR Action Spectroscopy Experiments with Sodium Doped Water Clusters. Phys. Chem. Chem. Phys. 2021, 23, 7682–7695. 10.1039/D0CP05390B. 33496289

[ref32] Kondow T.; Nagata T.; Kuchitsu K. A Mechanism of Electron Attachment to Small Clusters. Z. Phys. D - Atoms, Molecules and Cluster 1989, 12, 291–292. 10.1007/BF01426959.

[ref33] Lee S.-W.; Freivogel P.; Schindler T.; Beauchamp J. L. Freeze-Dried Biomolecules: FT-ICR Studies of the Specific Solvation of Functional Groups and Clathrate Formation Observed by the Slow Evaporation of Water from Hydrated Peptides and Model Compounds in the Gas Phase. J. Am. Chem. Soc. 1998, 120, 11758–11765. 10.1021/ja982075x.

[ref34] Schindler T.; Berg C.; Niedner-Schatteburg G.; Bondybey V. E. Protonated Water Clusters and Their Black Body Radiation Induced Fragmentation. Cheem. Phys. Lett. 1996, 250, 301–308. 10.1016/0009-2614(96)00002-4.

[ref35] Hansen K.; Näher U. Evaporation and Cluster Abundance Spectra. Phys. Rev. A 1999, 60, 1240 10.1103/PhysRevA.60.1240.

[ref36] Hansen K. Statistical Physics of Nanoparticles in the Gas Phase; Springer Series on Atomic, Optical, and Plasma Physics; Springer: Dordrecht, 2018; Vol. 73, 2nd ed.

[ref37] Niman J. W.; Kamerin B. S.; Kresin V. V.; Krohn J.; Signorell R.; Halonen R.; Hansen K. Shells in CO_2_ Clusters. Phys. Chem. Chem. Phys. 2022, 24, 5343–5350. 10.1039/D1CP05866E. 35191436

[ref38] Kazachenko S.; Thakkar A. J. Water nanodroplets: Predictions of five model potentials. J. Chem. Phys. 2013, 138, 194302 10.1063/1.4804399. 23697413

[ref39] Jongma R. T.; Huang Y.; Shi S.; Wodtke A. M. Rapid Evaporative Cooling Suppresses Fragmentation in Mass Spectrometry: Synthesis of “Unprotonated” Water Cluster Ions. J. Phys. Chem. A 1998, 102, 8847–8854. 10.1021/jp983366v.

[ref40] Dong F.; Heinbuch S.; Rocca J. J.; Bernstein E. R. Dynamics and Fragmentation of Van der Waals Clusters: (H_2_O)_n_, (CH_3_OH)_n_, and (NH_3_)_n_ Upon Ionization by a 26.5 eV Soft X-Ray Laser. J. Chem. Phys. 2006, 124, 224319 10.1063/1.2202314. 16784286

[ref41] Hansen K.; Andersson P. U.; Uggerud E. Activation energies for evaporation from protonated and deprotonated water clusters from mass spectra. J. Chem. Phys. 2009, 131, 124303 10.1063/1.3230111. 19791877

