# Supplementary material for: Metastable Evaporation of Molecules from Water Clusters
Source: J Phys Chem A. 2024 Sep 26;128(40):8679–89. doi: 10.1021/acs.jpca.4c04728 (PMC11648104; doi:10.1021/acs.jpca.4c04728)
Supplement: Supplementary file 1 — jp4c04728_si_001.pdf [file jp4c04728_si_001.pdf]

Supporting Information: Metastable Evaporation of Molecules from Water Clusters

Viktoriya Poterya,† Andrij Pysanenko,† Michal Fárník,*,† Juraj Fedor,*,† and Klavs Hansen*,‡

†*J. Heyrovský Institute of Physical Chemistry, v.v.i., Czech Academy of Sciences, Dolejškova 2155/3, 182 23 Prague, Czech Republic*

‡*Center for Joint Quantum Studies and Department of Physics, School of Science, Tianjin University, 92 Weijin Road, Tianjin 300072, China*

E-mail: michal.farnik@jh-inst.cas.cz; juraj.fedor@jh-inst.cas.cz; klavshansen@tju.edu.cn

Mass spectra

Table S1: Expansion conditions: T_R and T_N are reservoir and nozzle temperatures, respectiely; P_b is the buffer gas pressure, when it was introduced; $\bar{N}$ is the calculated neutral cluster mean size.

	buffer	P_b (bar)	T_R (°C)	T_N (°C)	$\bar{N}$
A)	–	–	100	130	50
B)	–	–	130	150	200
C)	Ar	2	100	130	–
D)	Ne	2	100	130	–
E)	–	–	150	160	530

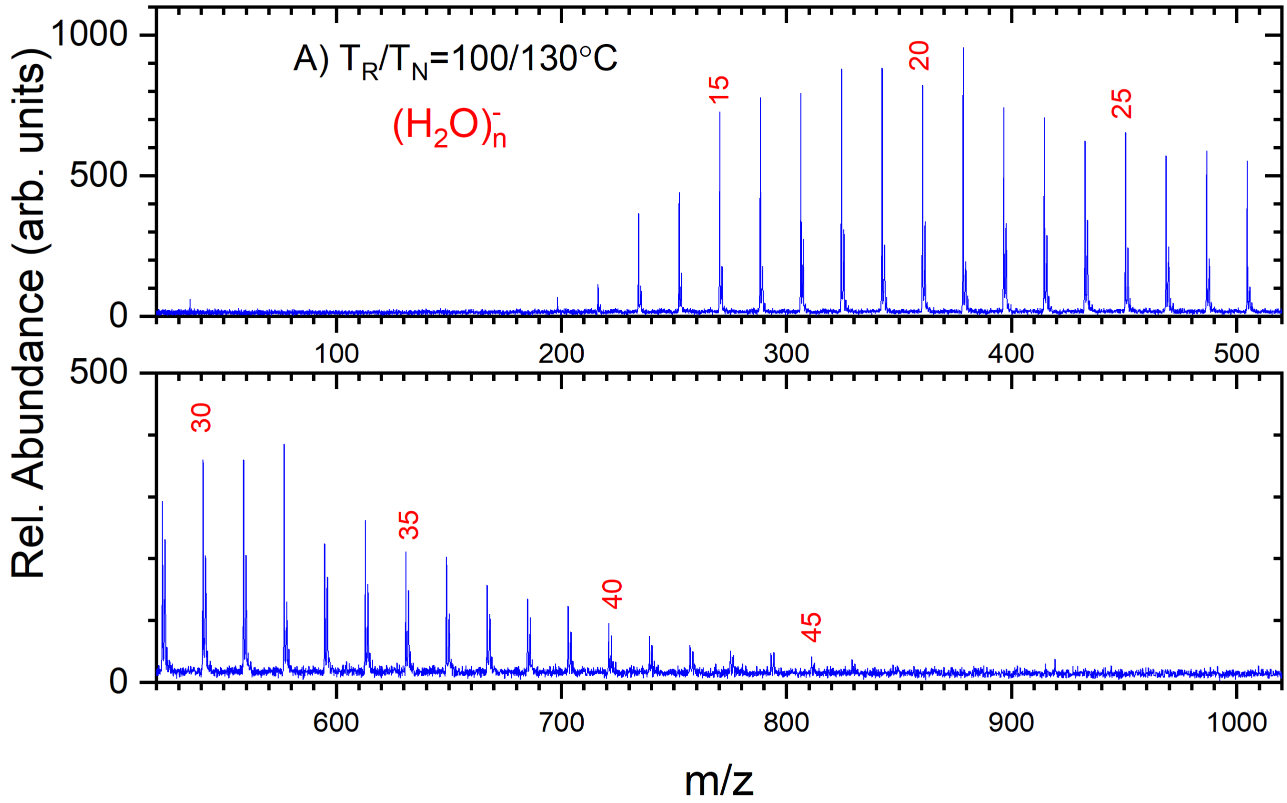

Figure S1: The negative ion mass spectrum, conditions A), Tab. S1

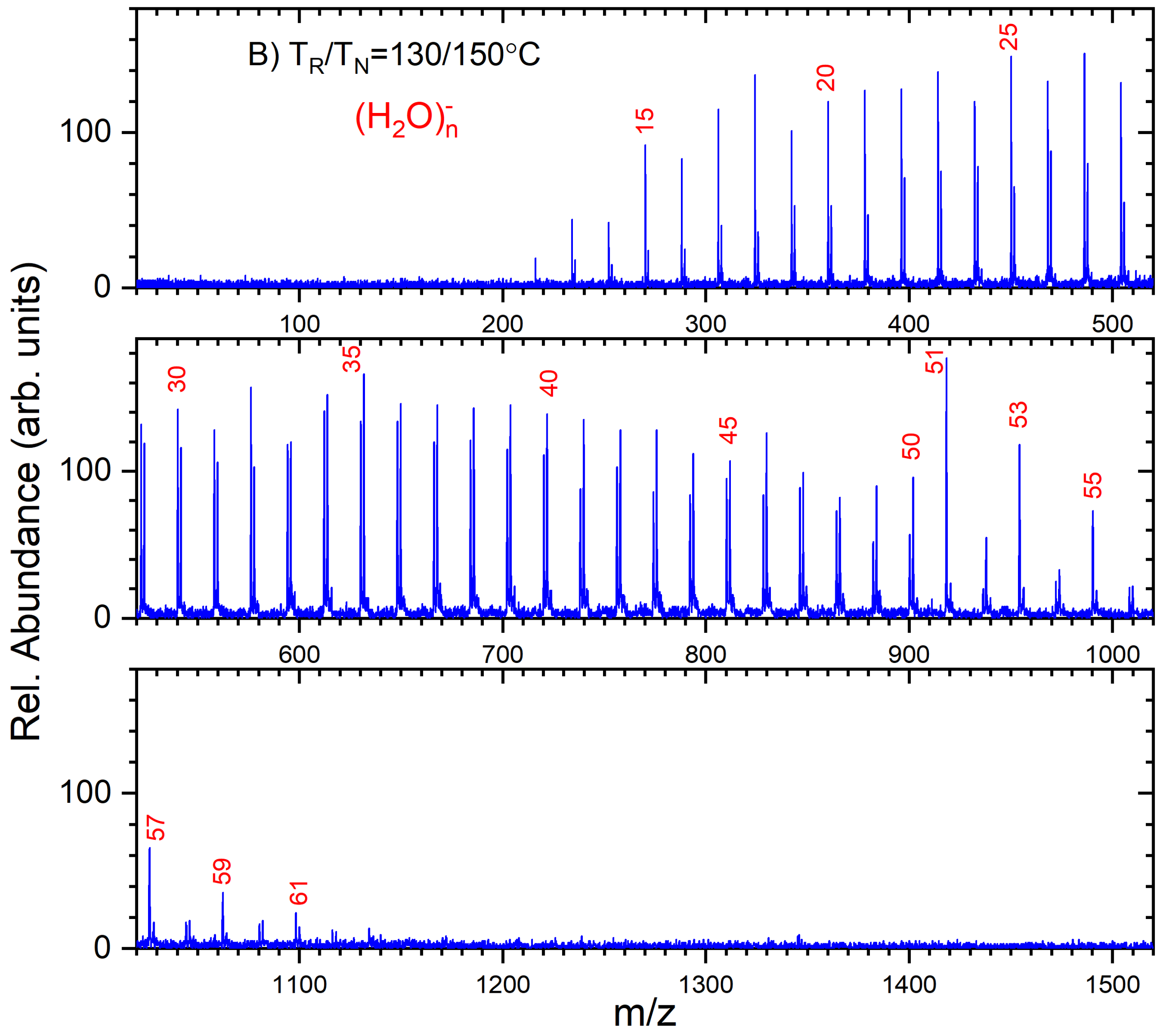

Figure S2: The negative ion mass spectrum, conditions B), Tab. S1

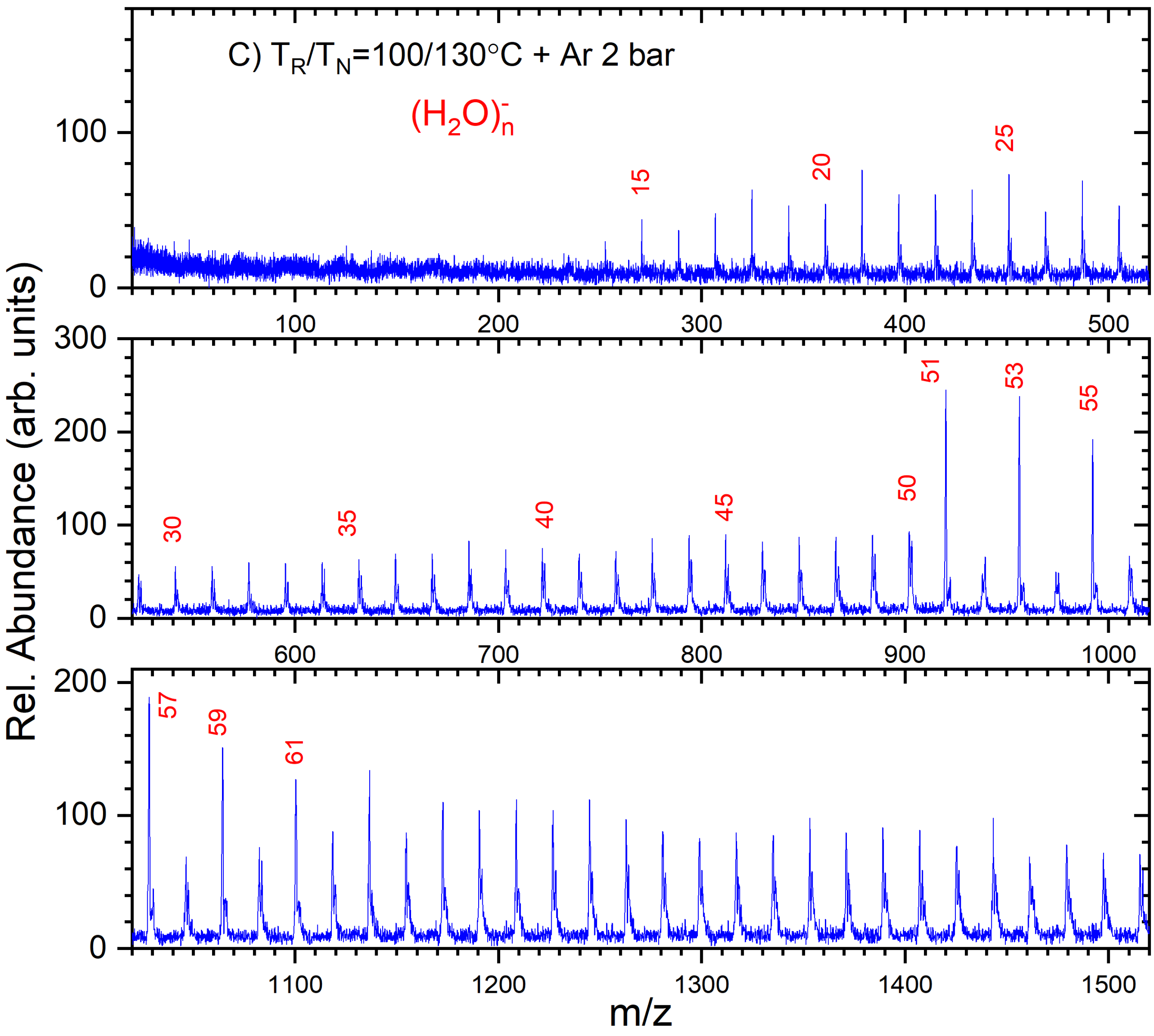

Figure S3: The negative ion mass spectrum, conditions C), Tab. S1

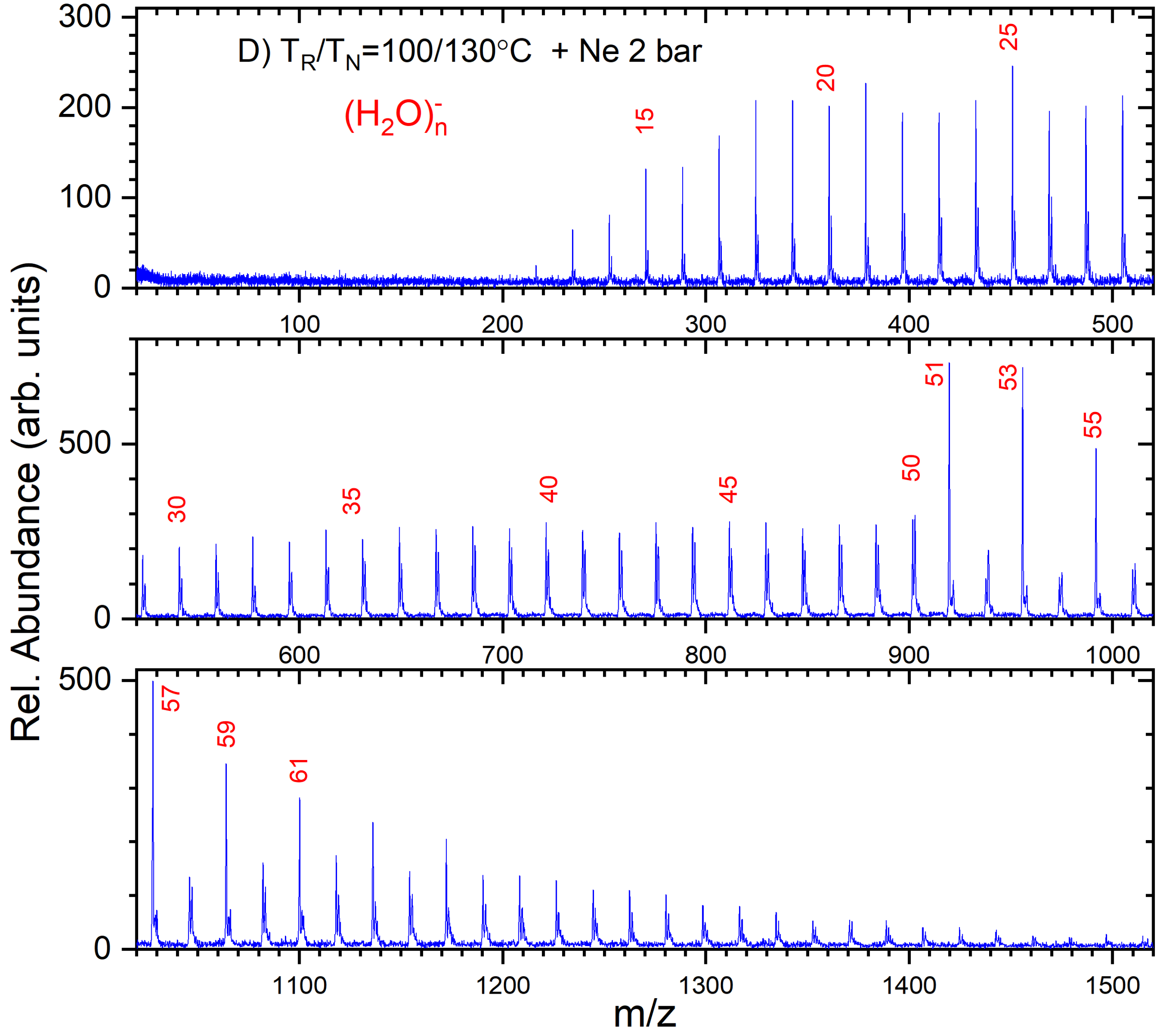

Figure S4: The negative ion mass spectrum, conditions D), Tab. S1

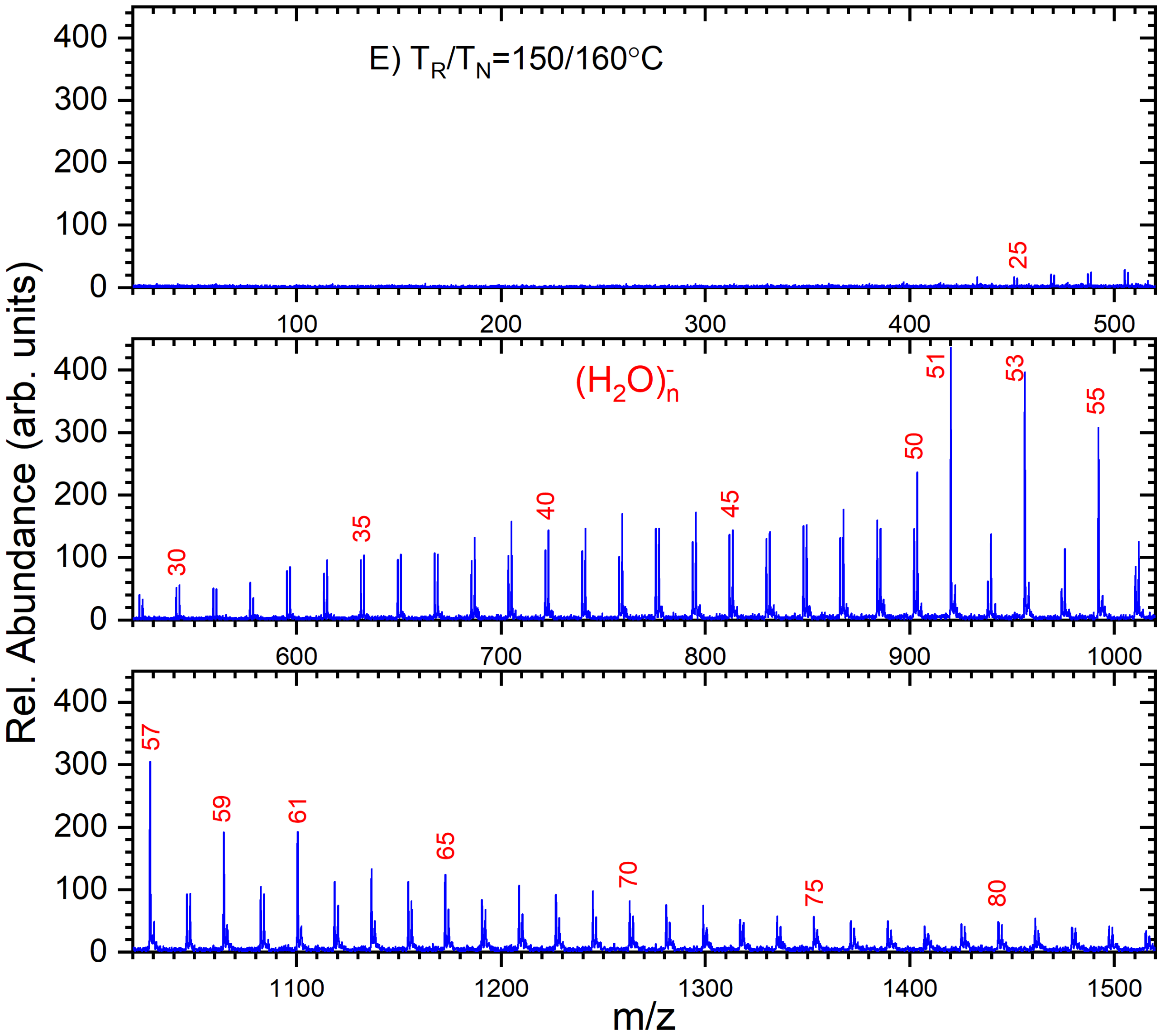

Figure S5: The negative ion mass spectrum, conditions E), Tab. S1
